# Short-Term Power Prediction of a Photovoltaic Power Station Based on the SSA-CEEMDAN-FCN Model

**DOI:** 10.1155/2022/6486876

**Published:** 2022-09-22

**Authors:** Zhaoyang Qu, Shaohua Qin, Genxin Xiong, Xinpo Zhu, Fan Ling, Yukun Wang, Juan Kong

**Affiliations:** ^1^School of Computer Science and Engineering & School of Software, Guangxi Normal University, Guilin 541004, China; ^2^Guangxi Key Lab of Multi-Source Information Mining and Security, Guangxi Normal University, Guilin 541004, China; ^3^Beijing China-power Puhua Co Ltd., Beijing 100085, China

## Abstract

Photovoltaic power generation is greatly affected by weather factors. To improve the prediction accuracy of photovoltaic power generation, complete ensemble empirical mode decomposition with an adaptive noise algorithm (CEEMDAN) is proposed to preprocess the power sequence. Then, the full convolutional network (FCN) model optimized based on the sparrow search algorithm (SSA) is used to predict the short-term photovoltaic power. SSA can more reasonably determine the parameters of FCN and improve the prediction performance of FCN. Therefore, the FCN model optimized by the SSA algorithm is used to establish prediction models for subsequences and predict each subsequence, respectively. Finally, the predicted value of each subsequence is superimposed. Taking the actual data of a photovoltaic power station in Jiangsu province of China as an example, by comparing some different common prediction models, it is proved that the proposed method is reasonable and feasible.

## 1. Introduction

With the increase in power demand, the use of fossil energy has a bad impact on the environment, resulting in huge global climate change. Therefore, a large number of people develop renewable energy. Therefore, accurate short-term photovoltaic power prediction can effectively alleviate the pressure of photovoltaic grid connection on the power system. Therefore, it is urgent to put forward an accurate prediction model, which is of great significance to ensure the stable operation of the power grid and the rational allocation of resources [1, 2].

Solar radiation and various meteorological factors make the prediction of photovoltaic power generation difficult. Therefore, to solve this problem, various prediction methods have been proposed. At present, they can be roughly divided into four categories: physical model, statistical model, machine learning model, and hybrid model:

1. A mathematical equation [3] is used to describe the physical state of the photovoltaic power generation system, which is classified as a physical model. When the weather is stable, the prediction accuracy of such models meets the demand, but when the weather changes violently, it cannot be predicted accurately [4, 5].
2. Common statistical models include an autoregressive moving average (ARMA) and its improvement [6–8]. Exponential smoothing and regression are two major categories in statistical models [9–13].
3. A machine learning model is developed based on a statistical model, but its prediction ability is stronger [14]. At present, the recurrent neural network (RNN) [15], limit learning machine (ELM) [16], support vector machine (SVM) [17], and other related models are commonly used in photovoltaic power generation prediction.

There are certain limitations in the separate prediction of the above three models. Therefore, by combining different prediction technologies, a hybrid model with higher prediction accuracy is designed. For example, the support vector machine (SVM) prediction of particle swarm optimization (PSO) based on wavelet transform (WT) [18] generates a mixture of the countermeasure network (GAN) and the convolutional neural network (CNN) [19].

At present, people usually analyze the relationship between the historical law of photovoltaic power data and external influencing factors, and thus, we establish a prediction model to predict the short-term photovoltaic power. However, at present, hybrid model prediction technology based on modal decomposition is a hot spot, and it cannot improve the prediction effect of photovoltaic power generation. For example, empirical mode decomposition (EMD) [20] decomposes the photovoltaic power sequence to reduce the nonstationary features of the photovoltaic power sequence so that the model can effectively extract subsequence features. EMD overcomes the defect that wavelet decomposition (WT) [21] requires human experience to select the basis function and decomposition levels in advance, but there is mode aliasing in the decomposition process. Ensemble empirical mode decomposition (EEMD) [22], complete ensemble empirical mode decomposition (CEEMD) [23], and CEEMDAN are improved methods of EMD, which eliminate mode aliasing in different ways. CEEMDAN shows good performance and is widely used in many fields [24–26]. In recent years, the application of deep learning in photovoltaic power prediction has achieved very remarkable results. A convolutional neural network (CNN) can learn the key features of local areas in the data sequence. Therefore, some researchers use CNN [27] as a feature extraction module to predict photovoltaic power generation in combination with LSTM. Some people also combined WT with a deep convolutional neural network (DCNN) and proposed a WT DCNN hybrid method for photovoltaic power generation prediction [28]. FCN is more flexible than CNN in processing time series data [29, 30]. Time series data can be input with any sequence length, and more sequence data features can be retained after FCN. For the hyperparametric optimization of neural networks, there are many optimization algorithms, but there is no experiment to prove that a certain algorithm is optimal. A genetic algorithm (GA) is a global optimization algorithm often used in the prediction model. The principle is to screen the population currently studied through the biological action mechanism and gradually select individuals with the highest fitness [31]. The principle of particle swarm optimization (PSO) is to use examples in the population to realize optimization by learning to continuously adjust the position and speed [32]. The sparrow search algorithm (SSA) is inspired by the sparrow's three behaviors of predation, tracking, and reconnaissance. According to the newly proposed prediction model, the sparrow search algorithm has good parameter optimization ability [33].

Therefore, this paper proposes a short-term power prediction model (SSA-CEEMDAN-FCN model) for photovoltaic power plants. The advantages of this model are as follows:

1. The photovoltaic power generation sequence is preprocessed by CEEMDAN. This improved data preprocessing method can well reduce the complexity of subsequence and improve the prediction performance of the model.
2. Considering the differences between subsequences, the key super parameters of the FCN model are optimized by SSA to obtain FCN with high prediction accuracy. The combination of SSA-FCN makes the FCN model play a better prediction performance.
3. Through the combination of CEEMDAN and SSA-FCN, a model with high prediction accuracy for the short-term power of the photovoltaic power station is obtained, which plays an important role in improving the utilization rate of photovoltaic power generation.

## 2. Methodology

### 2.1. Complete Ensemble Empirical Mode Decomposition with the Adaptive Noise Algorithm

Based on EMD, CEEMDAN overcomes the phenomenon of mode aliasing by adaptively adding Gaussian white noise and effectively decomposes nonstationary sequences. The specific steps of using CEEMDAN to decompose photovoltaic power sequence are as follows.

Let *Y* be the historical photovoltaic power sequence, *E*_*j*_(*∗*) be the *j*-th order modal component operator generated by EMD, *ω*_*n*_(*t*) be the Gaussian white noise sequence added for the *n*-th time, IMF be the *k*-th order eigenmode decomposition sequence obtained by CEEMDAN, and *δ*_*k*−1_ be the adaptive coefficient for solving *IMF*_*k*_.

(1) We add adaptive Gaussian white noise *δ*_0_*ωn*(*t*) to the original sequence *Y* and *n* = 1, 2,…, where *N* is the number of additions, i.e.,
  (1) $$\begin{array}{c} Y_{n}=Y+\delta_{0}\omega_{n}\left(t\right)\text{.} \end{array}$$
EMD decomposition is carried out for *Y*, respectively, so that *imf*_*n*_^1^ is the first-order modal component sequence of *Y*_*n*_ obtained by EMD. Then, the first-order eigenmode component sequence *IMF*_1_ and the first residual component sequence *r*_1_ of CEEMDAN decomposition are, respectively, as follows:
  (2) $$\begin{array}{c} IMF_{1}=N^{-1}\overset{N}{\underset{n=1}{\sum }}imf_{n}^{1},n=1,2,...,N\text{,}\\ r_{1}=Y-IMF_{1}\text{.} \end{array}$$
(2) We add adaptive Gaussian white noise *δ*_1_*E*_1_(*ω*_*n*_(*t*)) and *n* = 1, 2,…, *N* to the residual sequence *r*_1_, that is,
  (3) $$\begin{array}{c} r_{1n}=r_{1}+\delta_{1}E_{1}\left(\omega_{1}\left(t\right)\right)\text{.} \end{array}$$
EMD decomposition of *r*_1*n*_, respectively, then the second-order eigenmode component sequence *IMF*_2_ of CEEMDAN decomposition is as follows:
  (4) $$\begin{array}{c} IMF_{2}=N^{-1}\overset{N}{\underset{n=1}{\sum }}E_{1}\left(r_{1}+\delta_{1}E_{1}\left(\omega_{n}\left(t\right)\right)\right)\text{.} \end{array}$$
(3) For *k* = 2, 3,…, *K*, repeat step (2) to obtain the *k* + 1-order eigenmode component sequence and the *k*-th residual component sequence, namely,
  (5) $$\begin{array}{c} IMF_{2}=N^{-1}\overset{N}{\underset{n=1}{\sum }}E_{1}\left(r_{1}+\delta_{1}E_{1}\left(\omega_{n}\left(t\right)\right)\right)\text{,}\\ r_{k}=r_{k-1}+IMF_{k}\text{.} \end{array}$$
(4) Until the number of extreme points of the residual component sequence does not exceed 2, the final residual component sequence *R* is as follows:
  (6) $$\begin{array}{c} R=Y-\overset{K}{\underset{k=1}{\sum }}IMF_{k}\text{.} \end{array}$$
The historical photovoltaic power sequence *Y* is decomposed into *K* eigenmode component sequences *IMF*_*k*_ and a residual component sequence *R* by CEEMDAN, with a total of *K* + 1 subsequences.

### 2.2. Full Convolutional Neural Network

A convolutional neural network is widely used, but it can only solve the problem of fixed input size. To solve this problem, a full convolutional network is proposed. FCN has no full connection layer, which eliminates its restrictions on the shape of input data and the end-to-end training process of input and output. The processing of time series generally uses a one-dimensional CNN model. Therefore, this paper uses a one-dimensional FCN model to predict the power of a photovoltaic power station.

**Figure 1:**
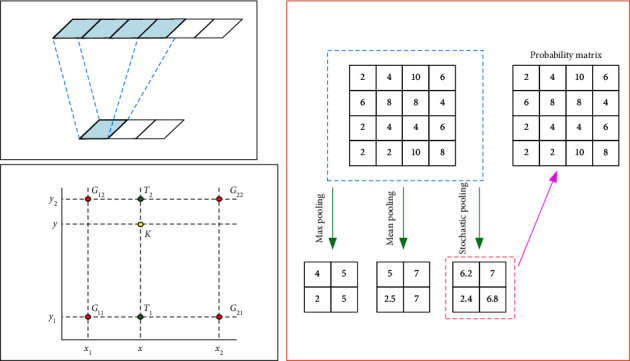
Convolution diagram.

(1) For the input data, the convolution kernel moves from left to right and from top to bottom, multiplies and sums each site covered by the convolution kernel with the convolution kernel, and finally obtains the output of this layer. The convolution process is shown in Figure 1(a). The convolution layer can extract the features of input data. In a full convolutional neural network, with the deepening of layers and the increase of the receptive field, it can extract the deep features of input data.
(2) The pooling layer of FCN is also known as the lower sampling layer. Using the pooling layer to remove redundant features in FCN can increase the receptive field, further reduce parameters, and prevent the network from overfitting. Figure 1(b) shows these three methods.
(3) Upper layer sampling is the convolution inverse process of the full convolutional network, which is used to enlarge the pooled characteristic map. FCN upper layer sampling uses the double line interpolation method. As shown in Figure 1(c), the values of function *F* in *G*_11_ = (*x*_1_, *y*_1_), *G*_12_ = (*x*_1_, *y*_2_), *G*_21_ = (*x*_2_, *y*_1_), and *G*_22_ = (*x*_2_, *y*_2_) are known, and the double line interpolation is used to obtain the value of at point *K* = (*x*, *y*). Therefore, the sampling process is completed by using four adjacent points in FCN.
  (7) $$\begin{array}{c} F\left(x,y_{1}\right)=\frac{x_{2}-x}{x_{2}-x_{1}}F\left(G_{11}\right)+\frac{x-x_{1}}{x_{2}-x_{1}}F\left(G_{21}\right)\text{,}\\ F\left(x,y_{1}\right)=\frac{x_{2}-x}{x_{2}-x_{1}}F\left(G_{12}\right)+\frac{x-x_{1}}{x_{2}-x_{1}}F\left(G_{22}\right)\text{,}\\ F\left(x,y\right)=\frac{y_{2}-y}{y_{2}-y_{1}}F\left(x,y_{1}\right)+\frac{y-y_{1}}{y_{2}-y_{1}}F\left(x,y_{2}\right)\text{.} \end{array}$$
(4) The traditional FCN network will make the prediction very rough after data processing in the convolution layer and pooling layer. The use of jump structure is to improve the accuracy of prediction.

### 2.3. Sparrow Search Algorithm

SSA divides sparrows into discoverers and accessors. The identities of discoverers and accessors in sparrows can be exchanged, but the overall proportion remains unchanged. The discoverer is responsible for searching for areas rich in food resources in the overall situation. The participants will monitor the discoverer and search for food or rob the discoverer's food in the area close to the discoverer. When the sparrow population finds danger, sparrows in the marginal area will quickly move closer to the safe area, and sparrows in the central area will move randomly. The steps of SSA are as follows:

- We initialize the sparrow population and define its relevant parameters, calculate and sort the fitness of all sparrows, find the sparrow with the best global fitness, and record its fitness value and its global optimal location.
- Iterate to update the location of the finder, entrant, and danger sensing sparrow. If the current global optimal fitness value is higher than the previous generation's optimal value, the update operation will be carried out. If not, the update will not be carried out and the iteration will continue.
- The fitness function converges or meets the conditions to obtain the global optimal value and the optimal fitness value.

**Figure 2:**
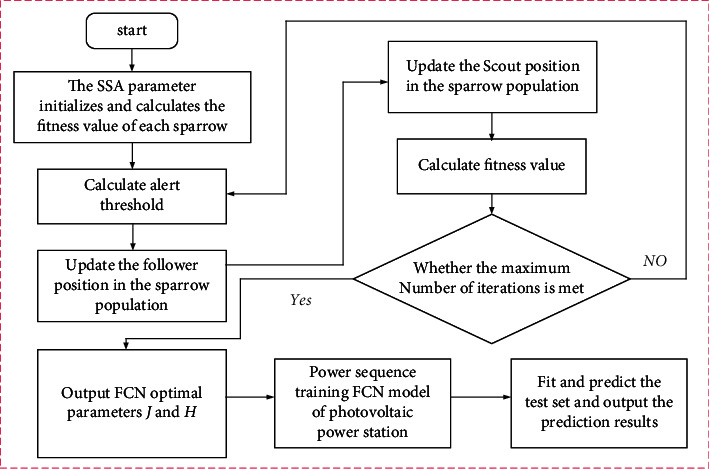
Flowchart of FCN optimized based on SSA.

Compared with other traditional optimization algorithms, it is easier to obtain the global optimal solution to the optimization problem. Therefore, SSA is used to optimize FCN, specifically as shown in Figure 2.

### 2.4. Structure Diagram of the SSA-CEEMDAN-FCN Model

**Figure 3:**
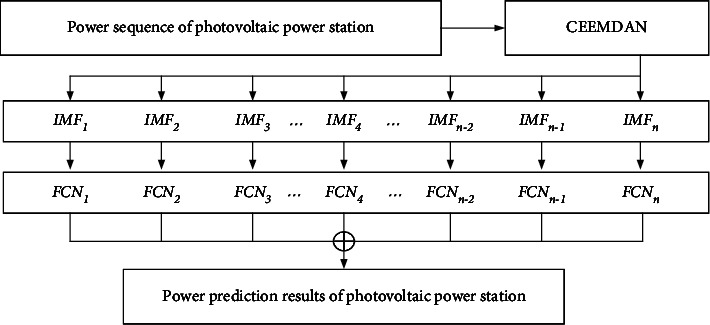
SSA-CEEMDAN-FCN prediction flowchart.

According to the previous basic theory, the overall framework flowchart based on the SSA-CEEMDAN-FCN combination model proposed in this study is shown in Figure 3. First, the power prediction sequence of the photovoltaic power station is preprocessed by CEEMDAN, and then, the decomposed subsequences are predicted by the SSA-optimized FCN model. Finally, the predicted values of subsequences are superimposed to obtain the final prediction results.

## 3. Actual Case Analysis and Verification

### 3.1. Data Description

**Figure 4:**
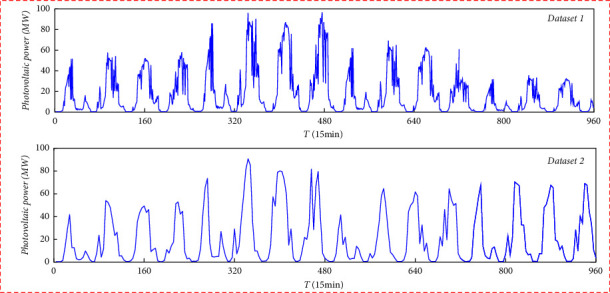
Photovoltaic power sequence diagram.

**Figure 5:**
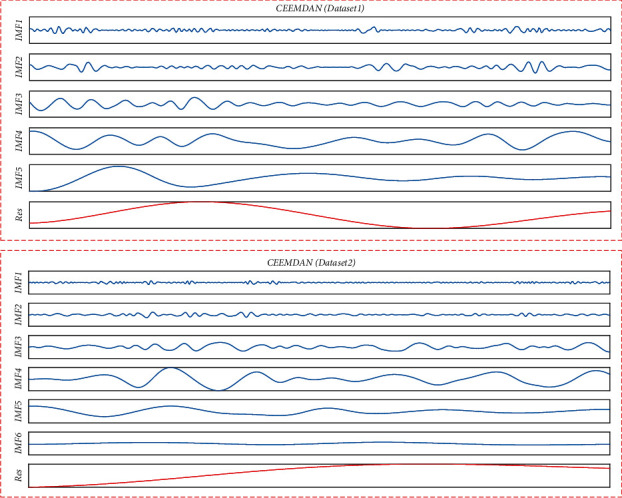
Photovoltaic power decomposition sequence.

Because this study belongs to the ultrashort-term prediction of photovoltaic power generation, from the measured data of photovoltaic power stations in a region of Jiangsu from 2019 to 2029, the data with a time length of 100 days are randomly selected for simulation experiments. Each experiment only needs to take 10 days for training and prediction, and the sampling period is 15 min. Two groups of continuous data with a length of 10 days are selected. The first 8 days of data are used as the training set of the model, the ninth day is used as the verification set, and the last 10 days of data are used as the test. The total installed capacity is 100 MW, and the time is from 06: 00 to 18: 00 every day. The 100-day photovoltaic power sequence and CEEMDAN decomposition sequence are shown in Figures 4 and 5, respectively, where the *x*-axis of Figure 5 represents the frequency of each subsequence.

It can be seen from Figures 4 and 5 that the subsequence of the CEEMDAN-decomposed sequence has more gentle characteristics. Dataset1 and dataset2 are decomposed into 5 and 6 subsequences, respectively, and then, there is a residual sequence, respectively. CEEMDAN can well reduce the complexity of the sequence.

### 3.2. Model Prediction and the Evaluation Index

This study uses two classical prediction and evaluation indexes, the mean absolute error (map) and the root mean square error (RMSE). The smaller the value of these two indicators, the better the performance of the model. Their expressions are as follows:

(8) $$\begin{array}{c} MAE=\frac{1}{n}\overset{n}{\underset{i=1}{\sum }}\left|Y_{i}-{\hat{Y}}_{i}\right|\text{,}\\ RMSE=\sqrt{\frac{1}{n}\overset{n}{\underset{i=1}{\sum }}{\left(Y_{i}-{\hat{Y}}_{i}\right)}^{2}}\text{,} \end{array}$$

where *n* is the predicted total number, *Y*_*i*_ is the actual photovoltaic power value in step *i*, and *Y*_*i*_ is the predicted value in step *i*.

### 3.3. Comparison of Prediction Results between Different Models

To reflect the superior prediction performance of the SSA-CEEMDAN-FCN model proposed in this study, a single model and their combined models will be used for prediction comparison. At present, the commonly used time series models with high prediction accuracy include ARIMA, LSTM, and ELM. The first 80% of the dataset is used as the training set, 80∼90% as the verification set, and the last 10% as the test set.

#### 3.3.1. Prediction of the Single Model in the Dataset

In dataset 1, the four single models ARIMA, LSTM, GRU, and FCN are compared for prediction.  Table 1 shows that compared with the other three single models, FCN has the highest prediction accuracy, and the prediction accuracy of the other three models is almost the same, but the prediction error is large.

#### 3.3.2. Comparison of Combined Models with the Decomposition Algorithm

Because the single model is a part of the combined model, it reflects the core prediction part of the combined model to a certain extent. For example, the prediction performance of the FCN model is the highest among several single models, but after the decomposition algorithm is added, the prediction accuracy of different models is improved by different percentages. Therefore, it is also necessary to verify whether the prediction performance of these combined models is improved and whether the prediction accuracy of the SSA-CEEMDAN-FCN model is the highest.

**Figure 6:**
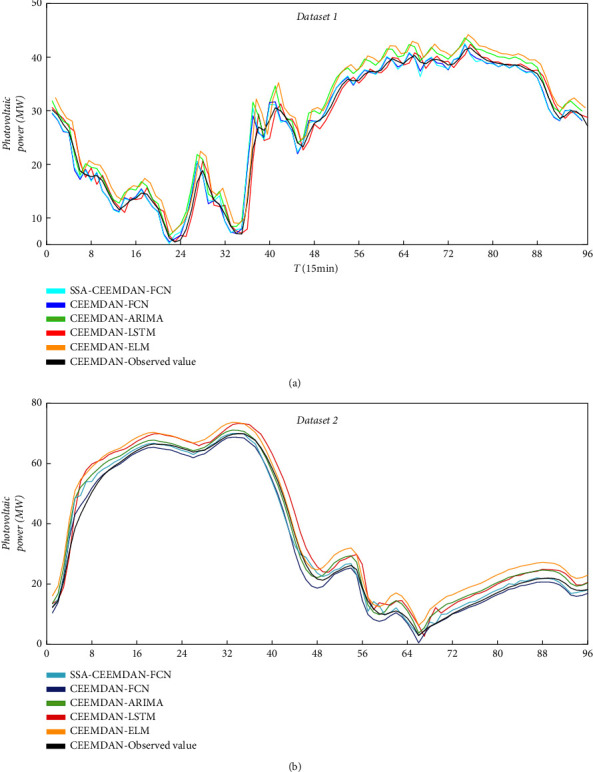
Comparison of prediction results of combined models of datasets 1 and 2.

It can be concluded that the prediction curve of the SSA-CEEMDAN-FCN model is the closest to the real observation value, and the prediction index of the SSA-CEEMDAN-FCN model is the smallest in both groups of data, indicating that its prediction accuracy is the highest. At the same time, we can conclude that after adding the CEEMDAN decomposition algorithm, the prediction accuracy of the combined model will be improved compared with that of the single model. Therefore, it also proves the superiority of the combined model. Therefore, the method proposed in this paper can better predict the changing trend of short-term photovoltaic power on the whole, and the prediction error is small. For example, in dataset 2, the prediction indexes RMSE and Mae of SSA-CEEMDAN-FCN are 2.32 and 1.47, respectively, which are far less than those of the other three combined models. The details are shown in Table 2 and Figure 6.

#### 3.3.3. FCN Model Prediction Performance under Different Optimization Algorithms

If only one parameter optimization method is used in the experiment, it will have the disadvantage of insufficient persuasion. Therefore, here, we use several other common classical optimization algorithms to optimize the model and compare their prediction results to prove the rationality of using the SSA method in this paper. The three comparison methods are the genetic optimization algorithm (GA), black hole optimization algorithm (BHA), and gray wolf optimization algorithm (GWO). Their comparison results in the two groups of data are shown in Table 3.

Therefore, from Table 3, we can see that the FCN model optimized by the FCN algorithm has the highest prediction accuracy, so the SSA optimization algorithm is used in the FCN network in the combined model proposed in this paper, which is very reasonable.

## 4. Conclusion

1. The photovoltaic power sequence is decomposed into subsequences with different frequencies by CEEMDAN, which can reduce the complexity of photovoltaic power and is conducive to power characteristic analysis, modeling, and prediction.
2. The FCN model optimized based on the sparrow search algorithm has better prediction performance, because the performance of SSA-CEEMDAN-FCN is better than that of the CEEMDAN-FCN model.
3. The SSA-CEEMDAN-FCN model is a combination model with excellent performance. At present, the prediction accuracy of LSTM and ELM models and their combined models with high prediction accuracy is lower than that of the SSA-CEEMDAN-FCN model. Therefore, the SSA-CEEMDAN-FCN model combined with the FCN model further improves the prediction accuracy of photovoltaic power.

The decomposition subsequence of photovoltaic power has different characteristic changes, and the prediction method of its higher performance needs to be further studied. Therefore, the photovoltaic power prediction will be further studied from these two aspects in the future.

## Figures and Tables

**Table 1:** Single model prediction performance versus results.

Dataset	Evaluating indicator (MW)	FCN	ARIMA	LSTM	ELM
1	MAE	6.57	9.34	9.21	8.24
	RMSE	7.20	9.21	8.76	8.61

2	MAE	5.47	9.87	8.87	8.17
	RMSE	6.32	10.01	9.76	9.55

**Table 2:** Results of combined model prediction performance.

Dataset	Evaluating indicator (MW)	SSA-CEEMDAN-FCN	CEEMDAN-FCN	CEEMDAN-ARIMA	CEEMDAN-LSTM	CEEMDAN-ELM
1	MAE	2.57	3.47	5.34	5.21	4.24
	RMSE	3.20	4.10	5.21	4.76	4.61

2	MAE	1.47	3.27	5.87	4.87	4.17
	RMSE	2.32	4.22	6.01	5.76	5.55

**Table 3:** Comparison of different optimization algorithms.

Dataset	Evaluating indicator (MW)	FCN (SSA)	FCN (GA)	FCN (BHA)	FCN (GWO)
1	MAE	6.57	7.38	9.19	7.99
	RMSE	7.20	8.11	8.63	8.43

2	MAE	5.47	9.64	7.84	8.11
	RMSE	6.32	9.87	8.33	9.35

## Data Availability

The dataset can be accessed upon request.
